# 2-Amino-5-nitro­benzoic acid

**DOI:** 10.1107/S1600536812002474

**Published:** 2012-01-25

**Authors:** Hakkı Yasin Odabaşoğlu, Orhan Büyükgüngör, Osman Ozan Avinç, Mustafa Odabaşoğlu

**Affiliations:** a Department of Textile Engineering, Faculty of Engineering, Pamukkale University, TR-20070 Kınıklı Denizli, Turkey; b Department of Physics, Faculty of Arts & Science, Ondokuz Mayıs University, TR-55139 Kurupelit Samsun, Turkey; c Department of Chemical Technonolgy, Pamukkale University, TR-20070 Kınıklı Denizli, Turkey

## Abstract

In the title compound, C_7_H_6_N_2_O_4_, an intra­molecular N—H⋯O hydrogen bond generates an *S*(6) ring. In the crystal, inversion dimers linked by pairs of O—H⋯O hydrogen bonds generate *R*_2_^2^(8) loops. Inter­molecular N—H⋯O and C—H⋯O hydrogen bonds then link the dimers, generating *R*_3_^3^(16)*R*_2_^1^(6) motifs. The whole mol­ecule is essentially planar, with the greatest deviation from the mean plane being 0.065 (2) Å.

## Related literature

For related structures of carb­oxy­lic acides, see: Mrozek & Glowiak (2004 ▶); Raza *et al.* (2010 ▶); Grabowski & Krygowski (1985 ▶). For hydrogen-bond motifs, see: Bernstein *et al.* (1995 ▶). For general background to *o*-amino­carb­oxy­lic acids, see: Fierz *et al.* (1949 ▶); Shore (2002 ▶).
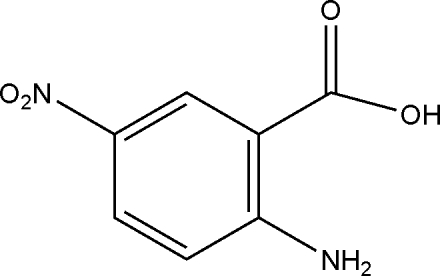


## Experimental

### 

#### Crystal data

C_7_H_6_N_2_O_4_
*M* *_r_* = 182.14
Monoclinic, 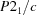

*a* = 3.7026 (3) Å
*b* = 17.4638 (16) Å
*c* = 11.6953 (10) Å
β = 92.210 (7)°
*V* = 755.67 (11) Å^3^
*Z* = 4
Mo *K*α radiation
μ = 0.13 mm^−1^
*T* = 296 K
0.55 × 0.23 × 0.06 mm

#### Data collection

Stoe IPDS II diffractometer
Absorption correction: integration (*X-RED32*; Stoe & Cie, 2002 ▶) *T*_min_ = 0.964, *T*_max_ = 0.992
5176 measured reflections
1567 independent reflections
884 reflections with *I* > 2σ(*I*)
*R*_int_ = 0.077

#### Refinement

*R*[*F* ^2^ > 2σ(*F* ^2^)] = 0.064
*wR*(*F* ^2^) = 0.127
*S* = 0.99
1567 reflections
118 parameters
H-atom parameters constrained
Δρ_max_ = 0.18 e Å^−3^
Δρ_min_ = −0.15 e Å^−3^

### 

Data collection: *X-AREA* (Stoe & Cie, 2002 ▶); cell refinement: *X-AREA*; data reduction: *X-RED32* (Stoe & Cie, 2002 ▶); program(s) used to solve structure: *SHELXS97* (Sheldrick, 2008 ▶); program(s) used to refine structure: *SHELXL97* (Sheldrick, 2008 ▶); molecular graphics: *ORTEP-3 for Windows* (Farrugia, 1997 ▶); software used to prepare material for publication: *WinGX* (Farrugia, 1999 ▶).

## Supplementary Material

Crystal structure: contains datablock(s) I, global. DOI: 10.1107/S1600536812002474/fk2050sup1.cif

Structure factors: contains datablock(s) I. DOI: 10.1107/S1600536812002474/fk2050Isup2.hkl

Supplementary material file. DOI: 10.1107/S1600536812002474/fk2050Isup3.cml

Additional supplementary materials:  crystallographic information; 3D view; checkCIF report

## Figures and Tables

**Table 1:** Hydrogen-bond geometry (Å, °)

*D*—H⋯*A*	*D*—H	H⋯*A*	*D*⋯*A*	*D*—H⋯*A*
N1—H7⋯O1	0.86	2.06	2.694 (3)	130
N1—H7⋯O4^i^	0.86	2.47	3.030 (3)	123
N1—H8⋯O3^ii^	0.86	2.39	3.192 (4)	155
O2—H2⋯O1^iii^	0.82	1.81	2.631 (3)	174
C6—H6⋯O3^ii^	0.93	2.54	3.347 (4)	145 (3)

Symmetry codes: (i) 
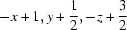
; (ii) 
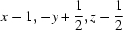
; (iii) 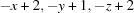.

**Fig. 1.**
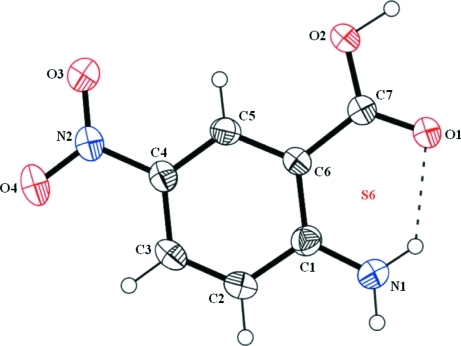
Molecular structure of (I). Anisotropic displacement ellipsoids are drawn at the 40% probability level.

**Fig. 2.**
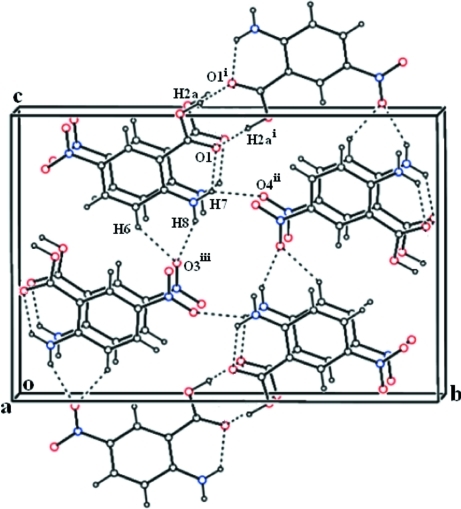
Crystal packing of (I), with hydrogen bonds drawn as dashed lines. [Symmetry codes: (i)x + 1/2, 1 - y, 2 - z; (ii)x - 1/2, y + 1/2, 3/2 - z; (iii)1/2 - x, 1/2 - y, z - 1/2].

## supplementary crystallographic information

### Comment

Most dyes contain groups known as auxochromes (colour helpers), examples of which are carboxylic acid, sulfonic acid, amino, and hydroxyl groups. While these groups are not responsible for colour, their presence can shift the colour of a colourant and they are most often used to influence dye solubility. Aminocarboxylic acids dissolve as easily in carbonate solution as does benzoic acid, and as easily in aqueous hydrochloric acid as does aniline. *o*-Aminocarboxylic acids are used for synthesis of azo dyes and indigo dyes (Fierz *et al.*, 1949; Shore, 2002). Functional groups such as carboxylic acids are completely inert in the reaction conditions for the azo coupling reaction. Taking into account these important features of the *o*-aminocarboxylic acids for the dye synthesis, we have undertaken the X-ray diffraction study of the 2-amino-5-nitrobenzoic acid, (I) (Fig. 1), in order to understand the molecular features which stabilize its observed conformation.

In previous works, 5-amino-2-nitrobenzoic acid (Mrozek & Glowiak, 2004), 2-Methylamino-5-nitrobenzoic acid (Raza *et al.*, 2010), 2,5-dinitrobenzoic acid (Grabowski & Krygowski, 1985) have been published whose molecular structures are similar to the title compound.

(I) is essentially planar, the largest deviation from the mean plane being -0.065 (2) Å for atom O1. The crystal packing is stabilized by N-H···O, O-H···O and C-H···O hydrogen bonds. There exists an S(6) ring motif (Bernstein *et al.*, 1995) due to the N-H···O intramolecular bond. Molecules are connected by intermolecular O-H···O hydrogen bonds to form centrosymmetric dimers with *R*_2_^2^(8) ring motifs. Other hydrogen bonds generate *R*_3_^3^(16)*R*_2_^1^(6) motifs (Fig. 2 and Table 1).

### Experimental

Yellow needles of 2-amino-5-nitrobenzoic acid were obtained by slow evaporation of the analytical reagent (Alfa Aesar) from ethyl alcohol solution (m.p. 543 K).

### Refinement

The H(O) position was derived from Fourier maps (HFIX 147), other H atoms were positioned geometrically and all were constrained to ride on their parent atoms, with 0.86 Å for N—H, 0.93 Å for aromatic C-H and 0.82 Å for O-H. The *U*_iso_(H) = *xU*_eq_(C/N), where *x* = 1.2 for H(N,C) and *x* = 1.5 for H(O).

### Crystal data

**Table:**

C_7_H_6_N_2_O_4_	*F*(000) = 376
*M**_r_* = 182.14	*D*_x_ = 1.601 Mg m^−^^3^
Monoclinic, *P*2_1_/*c*	Mo *K*α radiation, λ = 0.71073 Å
Hall symbol: -P 2ybc	Cell parameters from 4323 reflections
*a* = 3.7026 (3) Å	θ = 1.7–28.0°
*b* = 17.4638 (16) Å	µ = 0.13 mm^−^^1^
*c* = 11.6953 (10) Å	*T* = 296 K
β = 92.210 (7)°	Needle, orange
*V* = 755.67 (11) Å^3^	0.55 × 0.23 × 0.06 mm
*Z* = 4	

### Data collection

**Table:**

Stoe IPDS II diffractometer	1567 independent reflections
Radiation source: fine-focus sealed tube	884 reflections with *I* > 2σ(*I*)
graphite	*R*_int_ = 0.077
Detector resolution: 6.67 pixels mm^-1^	θ_max_ = 26.5°, θ_min_ = 2.1°
rotation method scans	*h* = −4→4
Absorption correction: integration (*X-RED32*; Stoe & Cie, 2002)	*k* = −21→21
*T*_min_ = 0.964, *T*_max_ = 0.992	*l* = −13→14
5176 measured reflections	

### Refinement

**Table:**

Refinement on *F*^2^	Primary atom site location: structure-invariant direct methods
Least-squares matrix: full	Secondary atom site location: difference Fourier map
*R*[*F*^2^ > 2σ(*F*^2^)] = 0.064	Hydrogen site location: geom and difmap
*wR*(*F*^2^) = 0.127	H-atom parameters constrained
*S* = 0.99	*w* = 1/[σ^2^(*F*_o_^2^) + (0.0486*P*)^2^] where *P* = (*F*_o_^2^ + 2*F*_c_^2^)/3
1567 reflections	(Δ/σ)_max_ < 0.001
118 parameters	Δρ_max_ = 0.18 e Å^−^^3^
0 restraints	Δρ_min_ = −0.15 e Å^−^^3^

### Special details

**Table:**

Geometry. All esds (except the esd in the dihedral angle between two l.s. planes) are estimated using the full covariance matrix. The cell esds are taken into account individually in the estimation of esds in distances, angles and torsion angles; correlations between esds in cell parameters are only used when they are defined by crystal symmetry. An approximate (isotropic) treatment of cell esds is used for estimating esds involving l.s. planes.
Refinement. Refinement of *F*^2^ against ALL reflections. The weighted *R*-factor *wR* and goodness of fit *S* are based on *F*^2^, conventional *R*-factors *R* are based on *F*, with *F* set to zero for negative *F*^2^. The threshold expression of *F*^2^ > σ(*F*^2^) is used only for calculating *R*-factors(gt) *etc*. and is not relevant to the choice of reflections for refinement. *R*-factors based on *F*^2^ are statistically about twice as large as those based on *F*, and *R*- factors based on ALL data will be even larger.

### Fractional atomic coordinates and isotropic or equivalent isotropic displacement parameters (Å^2^)

**Table:**

	*x*	*y*	*z*	*U*_iso_*/*U*_eq_	
C1	0.6059 (8)	0.35123 (17)	0.7289 (3)	0.0393 (7)	
C2	0.7771 (8)	0.34418 (16)	0.8394 (2)	0.0346 (7)	
C3	0.8661 (8)	0.27231 (16)	0.8809 (3)	0.0370 (7)	
H3	0.9810	0.2678	0.9527	0.044*	
C4	0.7890 (8)	0.20744 (16)	0.8187 (3)	0.0380 (7)	
C5	0.6210 (9)	0.21311 (18)	0.7098 (3)	0.0444 (8)	
H5	0.5688	0.1692	0.6673	0.053*	
C6	0.5351 (9)	0.28294 (17)	0.6667 (3)	0.0431 (8)	
H6	0.4261	0.2862	0.5939	0.052*	
C7	0.8660 (8)	0.41190 (17)	0.9097 (2)	0.0383 (7)	
N1	0.5107 (8)	0.41812 (15)	0.6824 (2)	0.0548 (8)	
H8	0.4074	0.4196	0.6154	0.066*	
H7	0.5526	0.4599	0.7195	0.066*	
N2	0.8912 (8)	0.13354 (14)	0.8636 (2)	0.0502 (7)	
O1	0.7787 (6)	0.47709 (12)	0.88101 (17)	0.0513 (6)	
O2	1.0433 (6)	0.39701 (12)	1.00684 (18)	0.0528 (7)	
H2	1.0837	0.4371	1.0415	0.079*	
O3	1.0514 (7)	0.12980 (13)	0.9571 (2)	0.0681 (8)	
O4	0.8171 (9)	0.07683 (14)	0.8068 (2)	0.0845 (10)	

### Atomic displacement parameters (Å^2^)

**Table:**

	*U*^11^	*U*^22^	*U*^33^	*U*^12^	*U*^13^	*U*^23^
C1	0.0370 (18)	0.0391 (17)	0.0415 (17)	0.0007 (14)	−0.0015 (13)	0.0015 (13)
C2	0.0378 (17)	0.0336 (16)	0.0325 (15)	−0.0016 (14)	−0.0002 (12)	−0.0043 (13)
C3	0.0391 (18)	0.0395 (16)	0.0321 (16)	−0.0021 (14)	−0.0016 (13)	−0.0019 (12)
C4	0.0395 (18)	0.0330 (16)	0.0416 (18)	−0.0020 (13)	0.0014 (13)	−0.0032 (13)
C5	0.048 (2)	0.0421 (19)	0.0429 (18)	−0.0067 (15)	−0.0033 (14)	−0.0124 (14)
C6	0.0469 (19)	0.047 (2)	0.0346 (17)	−0.0021 (15)	−0.0062 (14)	−0.0048 (13)
C7	0.0434 (19)	0.0348 (18)	0.0365 (16)	−0.0006 (14)	−0.0019 (14)	−0.0014 (13)
N1	0.080 (2)	0.0407 (15)	0.0426 (15)	0.0062 (15)	−0.0147 (14)	0.0012 (12)
N2	0.0641 (19)	0.0336 (15)	0.0525 (17)	−0.0013 (14)	−0.0031 (14)	−0.0037 (13)
O1	0.0766 (17)	0.0322 (12)	0.0440 (12)	−0.0002 (11)	−0.0120 (11)	−0.0020 (10)
O2	0.0786 (17)	0.0343 (11)	0.0440 (13)	−0.0030 (11)	−0.0187 (12)	−0.0061 (9)
O3	0.101 (2)	0.0436 (14)	0.0573 (15)	0.0040 (13)	−0.0228 (15)	0.0032 (11)
O4	0.134 (3)	0.0334 (14)	0.084 (2)	−0.0019 (15)	−0.0263 (18)	−0.0118 (13)

### Geometric parameters (Å, °)

**Table:**

C1—N1	1.330 (4)	C5—H5	0.9300
C1—C6	1.416 (4)	C6—H6	0.9300
C1—C2	1.423 (4)	C7—O1	1.227 (3)
C2—C3	1.381 (4)	C7—O2	1.316 (3)
C2—C7	1.470 (4)	N1—H8	0.8600
C3—C4	1.370 (4)	N1—H7	0.8600
C3—H3	0.9300	N2—O4	1.218 (3)
C4—C5	1.399 (4)	N2—O3	1.226 (3)
C4—N2	1.439 (4)	O2—H2	0.8200
C5—C6	1.353 (4)		
			
N1—C1—C6	119.3 (3)	C4—C5—H5	120.2
N1—C1—C2	123.3 (3)	C5—C6—C1	122.1 (3)
C6—C1—C2	117.4 (3)	C5—C6—H6	118.9
C3—C2—C1	119.3 (3)	C1—C6—H6	118.9
C3—C2—C7	119.3 (3)	O1—C7—O2	122.5 (3)
C1—C2—C7	121.4 (3)	O1—C7—C2	122.9 (3)
C4—C3—C2	121.5 (3)	O2—C7—C2	114.7 (3)
C4—C3—H3	119.2	C1—N1—H8	120.0
C2—C3—H3	119.2	C1—N1—H7	120.0
C3—C4—C5	120.1 (3)	H8—N1—H7	120.0
C3—C4—N2	120.1 (3)	O4—N2—O3	122.3 (3)
C5—C4—N2	119.8 (3)	O4—N2—C4	118.7 (3)
C6—C5—C4	119.5 (3)	O3—N2—C4	119.0 (3)
C6—C5—H5	120.2	C7—O2—H2	109.5
			
N1—C1—C2—C3	179.9 (3)	N1—C1—C6—C5	−179.1 (3)
C6—C1—C2—C3	0.1 (4)	C2—C1—C6—C5	0.8 (4)
N1—C1—C2—C7	−1.0 (4)	C3—C2—C7—O1	−176.2 (3)
C6—C1—C2—C7	179.1 (3)	C1—C2—C7—O1	4.8 (5)
C1—C2—C3—C4	−0.9 (4)	C3—C2—C7—O2	3.1 (4)
C7—C2—C3—C4	180.0 (3)	C1—C2—C7—O2	−176.0 (3)
C2—C3—C4—C5	1.0 (5)	C3—C4—N2—O4	179.5 (3)
C2—C3—C4—N2	178.9 (3)	C5—C4—N2—O4	−2.5 (5)
C3—C4—C5—C6	−0.1 (5)	C3—C4—N2—O3	−0.9 (5)
N2—C4—C5—C6	−178.1 (3)	C5—C4—N2—O3	177.0 (3)
C4—C5—C6—C1	−0.7 (5)		

### Hydrogen-bond geometry (Å, °)

**Table:**

*D*—H···*A*	*D*—H	H···*A*	*D*···*A*	*D*—H···*A*
N1—H7···O1	0.86	2.06	2.694 (3)	130
N1—H7···O4^i^	0.86	2.47	3.030 (3)	123
N1—H8···O3^ii^	0.86	2.39	3.192 (4)	155
O2—H2···O1^iii^	0.82	1.81	2.631 (3)	174
C6—H6···O3^ii^	0.93	2.54	3.347 (4)	145 (3)

Symmetry codes: (i) −*x*+1, *y*+1/2, −*z*+3/2; (ii) *x*−1, −*y*+1/2, *z*−1/2; (iii) −*x*+2, −*y*+1, −*z*+2.
